# Supplementary material for: Sorting at embryonic boundaries requires high heterotypic interfacial tension
Source: Nat Commun. 2017 Jul 31;8:157. doi: 10.1038/s41467-017-00146-x (PMC5537356; doi:10.1038/s41467-017-00146-x)
Supplement: Supplementary file 2 — Supplementary Software 1 [file 41467_2017_146_MOESM2_ESM.zip › PottsModel/SrcPottsModel/doc/index-files/index-18.html]

S-Index


JavaScript is disabled on your browser.


Skip navigation links


- Overview
- Package
- Class
- Use
- Tree
- Deprecated
- Index
- Help

- Prev Letter
- Next Letter

- Frames
- No Frames

- All Classes

A B C D E F G H I J K L M N O P R S T U V W 


## S

saveSnapshot(String) - Method in class gui.PottsCanvas


saveSnapshot(Lattice, String) - Method in class gui.SnapshotManager


scale(Color, double) - Static method in class utils.ColorScale


second - Variable in class model.Pair


setAngle(double) - Method in class model.HalfLine


setAutomaticMode(boolean) - Method in class gui.PottsFrame


setAutomaticMode(boolean) - Method in class gui.PottsToolbar


setAutomaticMode(boolean) - Method in class model.Constants


setCellCoordinatesCSVStatisticTracked(boolean) - Method in class model.Constants


setCellShapeCSVStatisticTracked(boolean) - Method in class model.Constants


setCellTypeInteractionEnergies(HashMap<EnumSet<Cell.CellType>, Double>) - Method in class model.Constants.ParameterManager


setColorAt(int, int, Color) - Method in class gui.PottsCanvas
:   Draw pixel at (x,y) with given color.

setDisplay(boolean) - Method in class gui.PlotPanel


setEdge(int, int, PixelShape.Edge, boolean) - Method in class gui.PottsCanvas
:   Draw pixel at (x,y) with given color.

setEnabled(boolean) - Method in class model.Constants


setGraphicalOutput(boolean) - Method in class model.Constants


setInitialLatticeFilename(String) - Method in class model.Constants


setInteractionEnergy(Cell.CellType, Cell.CellType, double) - Method in class model.Constants.ParameterManager


setMaxMcs(int) - Method in class model.Constants


setMCSButton(boolean) - Method in class gui.PottsToolbar


setOrigin(Point) - Method in class model.FiniteVector


setOrigin(Point) - Method in class model.HalfLine


setOrigin(Point) - Method in interface model.Vector


setPixelSize(int) - Method in class model.Constants


setPixelType(PixelShape.Type) - Method in class model.Constants


setPoints(Point, Point) - Method in class model.FiniteVector


setRandomCellTypes(boolean) - Method in class model.Constants


setRecorded(boolean) - Method in class engine.PottsEngine


setRepeatFrequency(int) - Method in class engine.Statistic


setRootDirectory(String) - Method in class model.Constants


setScalePixelSize(boolean) - Method in class model.Constants


setScreenHeight(int) - Method in class model.Constants


setScreenWidth(int) - Method in class model.Constants


setSimulationName(String) - Method in class model.Constants


setSnapshotSaved(boolean) - Method in class model.Constants


setSwapButton(boolean) - Method in class gui.PottsToolbar


setTarget(Point) - Method in class model.FiniteVector


setTracker(Utils.EnergyTracker) - Method in class engine.EnergyStatistic


setTracker(Utils.EnergyTracker) - Method in class engine.StatisticsManager


setTracker(Utils.EnergyTracker) - Method in class model.EnergyManager


setX(double) - Method in class gui.Hexagon.AxialCoordinates


setX(double) - Method in class gui.Hexagon.CubeCoordinates


setX(double) - Method in class gui.Hexagon.OffsetCoordinates


setX(double) - Method in class model.CartesianCoordinates


setX(double) - Method in interface model.Coordinates


setY(double) - Method in class gui.Hexagon.AxialCoordinates


setY(double) - Method in class gui.Hexagon.CubeCoordinates


setY(double) - Method in class gui.Hexagon.OffsetCoordinates


setY(double) - Method in class model.CartesianCoordinates


setY(double) - Method in interface model.Coordinates


setZ(double) - Method in class gui.Hexagon.AxialCoordinates


setZ(double) - Method in class gui.Hexagon.CubeCoordinates


setZ(double) - Method in class gui.Hexagon.OffsetCoordinates


setZ(double) - Method in class model.CartesianCoordinates


setZ(double) - Method in interface model.Coordinates


showGUI() - Method in class gui.PlotPanel


showInfo() - Method in class gui.PottsFrame


showStats() - Method in class gui.PottsFrame


Simulation - Class in engine


Simulation() - Constructor for class engine.Simulation


simulations - package simulations


SingleCell - Class in simulations


SingleCell() - Constructor for class simulations.SingleCell


size() - Method in class model.CellSet


SnapshotManager - Class in gui
:   Graphical representation of the Potts Model Lattice.

SnapshotManager(int, int, Constants) - Constructor for class gui.SnapshotManager
:   Constructs a Cartesian grid that can carry (pPottsWidth x pPottsHeight)
    PottsPixels a.k.a Spins.

Square - Class in gui


Square(int, int, int) - Constructor for class gui.Square


SquareCell - Class in model


SquareCell(Cell.CellType, int, Lattice) - Constructor for class model.SquareCell


SquareCell(int, Lattice) - Constructor for class model.SquareCell


SquareCell(SquareCell) - Constructor for class model.SquareCell


SquarePixelDisplay - Class in gui


SquarePixelDisplay(int, int, int, Color) - Constructor for class gui.SquarePixelDisplay


SquarePixelDisplay(int, int, int) - Constructor for class gui.SquarePixelDisplay


SquarePositionManager - Class in model


SquarePositionManager(Lattice) - Constructor for class model.SquarePositionManager


SquarePositions - Static variable in interface model.IPositionManager


StateVariables() - Constructor for class engine.PottsEngine.StateVariables


Statistic - Class in engine


Statistic(PottsEngine, String, int) - Constructor for class engine.Statistic


Statistic(PottsEngine, String) - Constructor for class engine.Statistic


Statistic.Utils - Class in engine


StatisticsManager - Class in engine
:   Manages statistics by initailizing and evaluating the Statistics according to the frequency
    defined by the user in the simulation settings file.

StatisticsManager(PottsEngine) - Constructor for class engine.StatisticsManager


StatusBar - Class in gui


StatusBar(PottsFrame) - Constructor for class gui.StatusBar


std(List<Double>) - Static method in class engine.Statistic.Utils


std(float[]) - Static method in class engine.Statistic.Utils


std(EnergyFunction.Energy) - Method in class engine.Utils.EnergyTracker

A B C D E F G H I J K L M N O P R S T U V W

Skip navigation links


- Overview
- Package
- Class
- Use
- Tree
- Deprecated
- Index
- Help

- Prev Letter
- Next Letter

- Frames
- No Frames

- All Classes
